# Renal involvement without infiltration of eosinophil in Kimura’s disease

**DOI:** 10.1080/0886022X.2020.1865170

**Published:** 2021-01-13

**Authors:** Ying Luo, Xiu-mei Hu, Jie Li, Hong-yan Li, Xiang-meng Yi, Qing-feng Peng

**Affiliations:** Department of Nephrology, Zhuzhou Central Hospital, Zhuzhou, Hunan, China

Dear Editor,

Kimura’s disease (KD), also known as eosinophilic lymphoid granuloma, is a rare and chronic inflammatory disorder of unknown causes, which has a predilection for involvement of young Asian males [1]. The clinical manifestations of this disease are painless subcutaneous soft tissue masses in the head and neck which grow slowly, and are accompanied by lymphadenopathy. Laboratory tests show elevation of eosinophilia and immunoglobulin E. KD follows a benign course, and no malignant transformation has been reported so far. However, the disease is easy to relapse [2]. In addition, due to lack of specificity in clinical manifestations, laboratory and imaging examinations, KD is easy to be misdiagnosed. Previous research has shown that the complication rate of renal involvement was 12% to 16% in patients with KD [3], and the most common features is eosinophilic infiltration in renal tissue [4,5]. In this paper, we described a case of nephrotic syndrome associated with KD without infiltration of eosinophil in the kidney.

A 16-year-old Chinese man was admitted with edema in both eyelids and legs and oliguria for 15 days on 28 August 2017. A mass was found in the right retroauricular maxillofacial region for more than 1 year without obvious change. There was no family history of renal diseases. Physical examinations showed high blood pressure at 138/84 mmHg, nephrotic facies, scattered rash on the face, mild edema on both eyelids and severe edema in both lower extremities. A 3cmx4cm rubbery mass without tender was found in the right retroauricular maxillofacial region, with intact skin and sharp boundary. Laboratory evaluations revealed normal white blood cell count of 5.69 × 10^9^/L (normal range: 4–10 × 10^9^/L), increasing eosinophilia count of 1.14 × 10^9^/L (normal range: 0.05–0.5 × 10^9^/L) and increasing percentage of eosinophils of 20.04% (normal range:0.5–5%). Urinalysis showed that albuminuria was +++ and 24-h urine total protein was 6.4 g, with serum albumin of 12.4 g/L (normal range: 40–55 g/L) and serum cholesterol of 13.36 mmol/L (normal range: 3.35–5.72 mmol/l). Renal function was normal. Antineutrophil cytoplasmic antibodies, antinuclear antibody, hepatitis B virus, hepatitis C virus, and human immunodeficiency virus were all negative. B-mode ultrasound indicated that mass in the right retroauricular maxillofacial region was swelling lymph nodes. On September 11, the mass was completely removed and was performed pathological examination, characterized by fibrous tissue proliferation with numerous eosinophilic infiltration in lymph nodes. Therefore, the patient was diagnosed with KD (Figure 1). Subsequently, this patient underwent renal biopsy on September 13, which indicated minimal change disease (MCD; Figure 2). Although there was no typical infiltration of eosinophil in the renal tissue, urinary output of the patient increased significantly two days after the mass excision (September 13), which was 1500–2000 mL per day without diuretics (500 mL per day at the time of admission, 1200–2000 mL per day with albumin infusion and diuretics).

**Figure 1.**
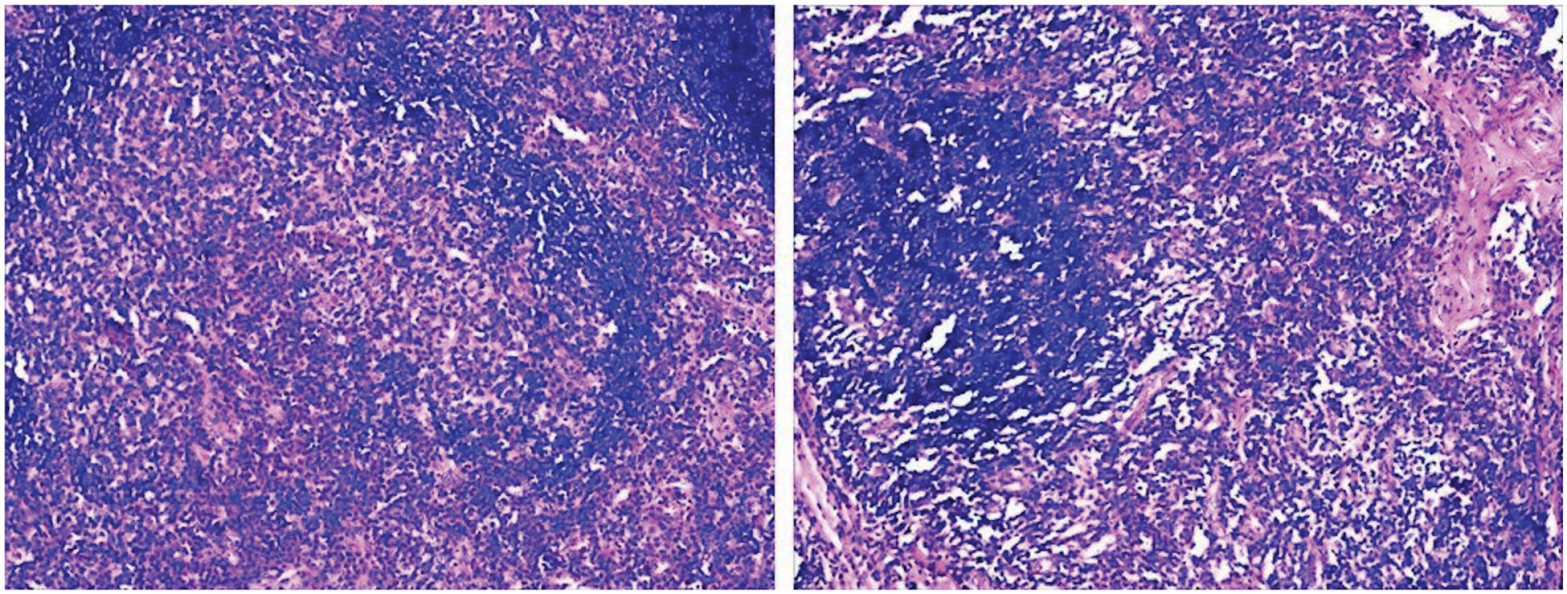
Histological examination of a lymph node biopsy, fibrous tissue proliferation with numerous eosinophilic infiltration in lymph nodes.

**Figure 2.**
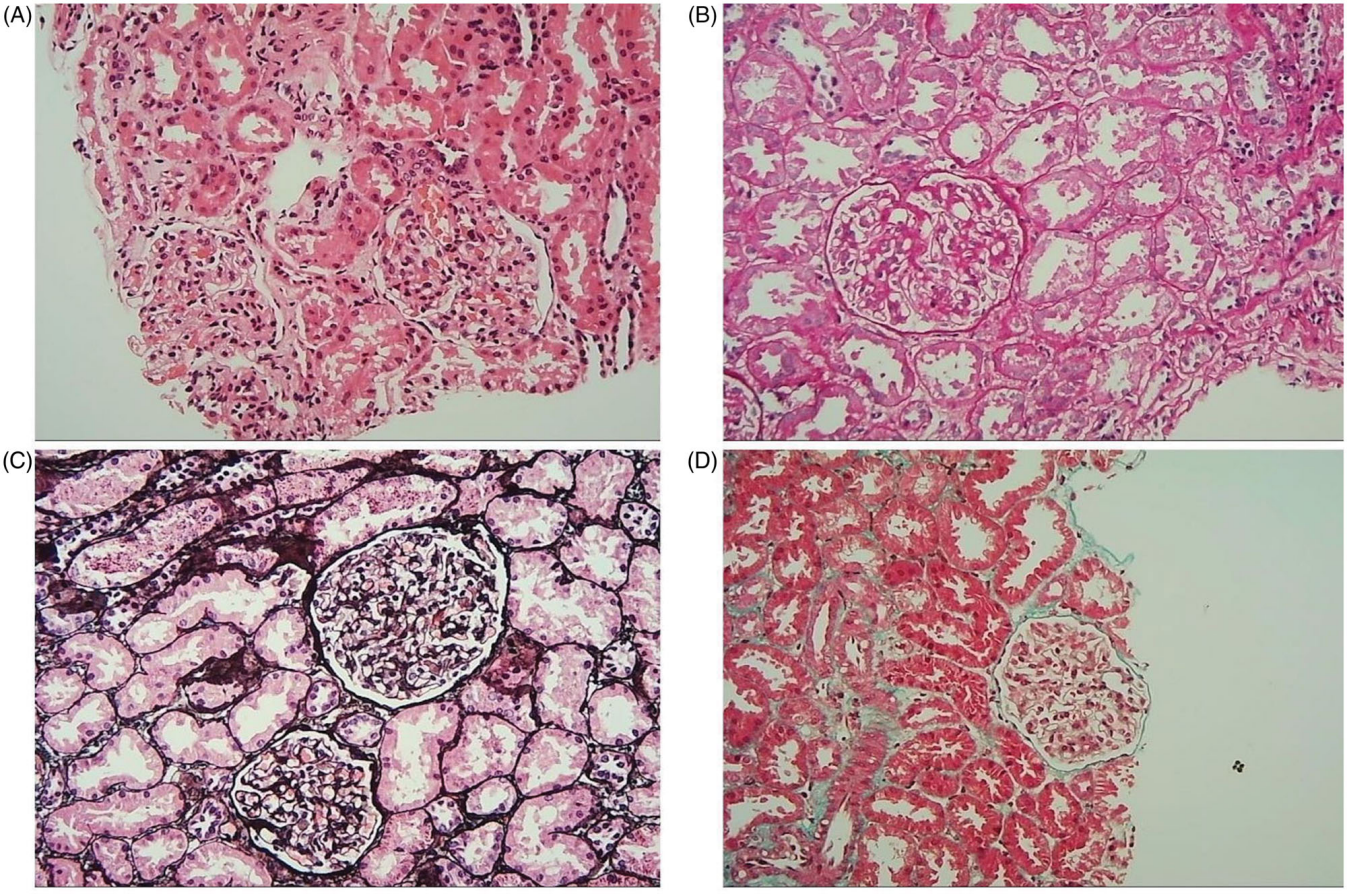
Histological examination of a renal biopsy showed minimal change disease (MCD) and there was no typical infiltration of eosinophil in the renal tissue. (A) The lesion shows mild in the glomerulus(H&E staining × 200). (B) Mesangial cells proliferation and mesangial matrix expansion (PAS staining × 200). (C) No thickening in glomerulus basement membrane and the glomerular capillary loops open well (PASM staining × 200). (D) There is no obvious immune complex deposition in the glomerulus (Mason staining × 200).

The patient was finally diagnosed as nephrotic syndrome (pathological type was MCD) associated with KD based on typical clinical manifestations and histopathological findings. He was given intravenous methylprednisolone (40 mg/day) for 3 days from 20 September 2017, followed by oral methylprednisolone (32 mg/d, once a day). Four days after the initiation of glucocorticoid treatment, edema was significantly relieved, eosinophilia count and the percentage of eosinophils returned to normal, serum albumin level improved, and 24-h urine total protein decreased to 1.39 g. The patient reached complete remission after 1.5 months with methylprednisolone treatment. After a 2-month period of methylprednisolone treatment at a dose of 32 mg/day, the dose was tapered to 28 mg/day, followed by further reduction of 4 mg every 4 weeks; when the dose reached 16 mg/day, methylprednisolone was tapered by 2 mg every 4 weeks. During 2 years follow-up, serum albumin levels, renal function and urinalysis test remained normal and no relapses occurred. No adverse reaction was observed.

Histopathological examination is the golden standard for diagnosis of KD, characterized by intact nodal structure with follicular hyperplasia and the proliferation of vessels surrounded by infiltrating lymphocytes and eosinophils. An eosinophilic microabscess was detected in the cortical area of the lymph nodes [6]. The histological features of renal involvement associated with KD are variable and nonspecific, including MCD, membranous nephropathy, membranoproliferative glomerulonephritis, IgA nephropathy, focal segmental glomerulosclerosis and so on [4,7]. Previous studies have suggested that the most common features is scattered or patchy distribution of eosinophil infiltration mainly around sclerotic glomeruli or interstitium [5,7,8]. Interestingly, the eosinophil infiltration in the renal tissue was not seen in all patients of KD with renal involvement, which suggest that renal damage is not only caused by direct infiltration of eosinophilic cells, but also involves other immune factors (such as TNF, IL, etc.) [6,8]. Currently, there is no golden standard for the diagnosis of renal involvement associated with KD, which is mainly based on the diagnosis of KD and the clinical manifestations of renal injury.

In short, for patients with proteinuria and hematuria, especially adolescent males, if there are lymphadenopathy in the head and neck combined with elevated peripheral eosinophils and serum IgE, KD is need to be considered. The renal biopsy and lymph node biopsy in the lesion should be conducted in time to make right diagnosis and guide treatment. Currently, the pathogenesis of KD with renal involvement has not been fully elucidated, its diagnostic criteria is not uniform and the therapy does not reache agreement. These problems remain to be further studied.

## Data Availability

All data supporting the case are included in the manuscript.
